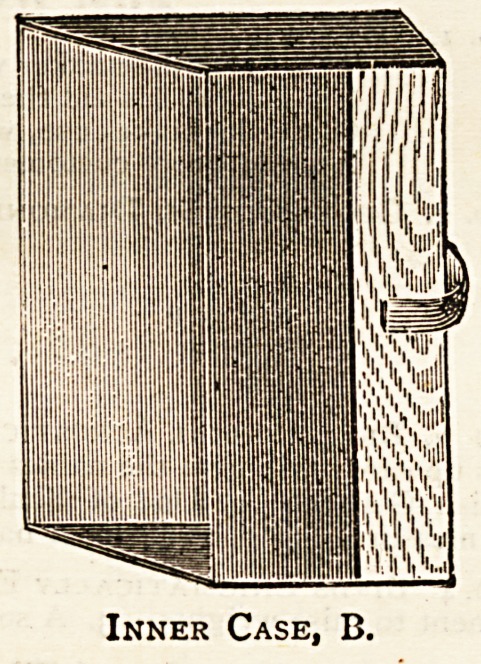# New Remedies and Appliances

**Published:** 1888-08-04

**Authors:** 


### New Remedies and Appliances.

The Marlborough Case.—Of the many contrivances which have been offered to the public in the name of convenience and order, none are simpler or neater than the Marlborough Pamphlet case, while few are so economical in price. As will be seen from the illustrations here given, it consists of a pocket to contain the pamphlets or papers, and has on its side a register for entering the numbers, names, dates, etc., of the papers. This pocket, or inner case, B, is slid into the outer case, A, which is made to fit it,

Outer Case, A. Inner Case, B.

and which is book-shaped, with a rounded back, bearing a label indicating the general contents of the case. We have no doubt these cases will become very popular, as they supply a means of preserving pamphlets from dust and destruction, and of keeping documents (which may be numbered at the left-hand top corner), not only free from dust, but ready for instantaneous reference. They will be a real boon to librarians, secretaries, clergymen, and indeed to all who are desirous of keeping unbound literature and other papers at once easily accessible, and *secure and tidy*. They may also be had in almost any size, from ordinary letter size to that of the *Graphic* and *Illustrated London News*. They vary in thickness, from one and a-half to three and a-half inches, and their cost is only a few pence at most. The manufacturers are Marlborough, Gould, and Co., Old Bailey, E.C.

## Figures and Tables

**Figure f1:**
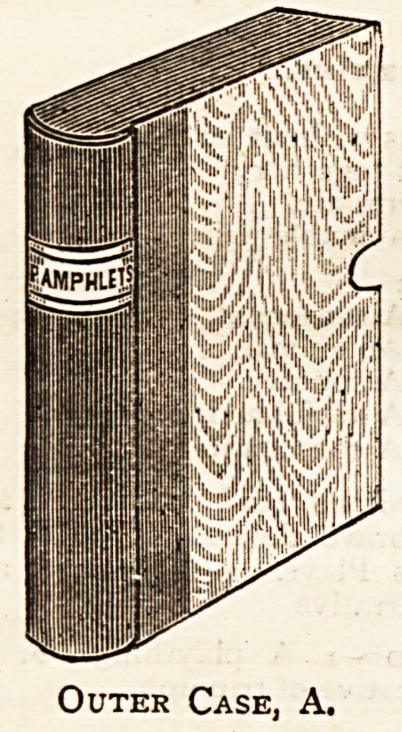


**Figure f2:**